# Lessons learned from the translation of the Internalised Stigma of Mental Illness (ISMI) scale into isiXhosa for use with South African Xhosa people with schizophrenia

**DOI:** 10.1177/13634615231168461

**Published:** 2023-06-18

**Authors:** Olivia P. Matshabane, Paul S. Appelbaum, Marlyn C. Faure, Patricia A. Marshall, Dan J. Stein, Jantina de Vries, Megan M. Campbell

**Affiliations:** 1 Department of Medicine, 63726 University of Cape Town, Cape Town, South Africa; 2 Social and Behavioral Research Branch, National Institutes of Health, Bethesda, MD, USA; 3 27424 New York State Psychiatric Institute, New York, NY, USA; 4 Department of Psychiatry, 5798 Columbia University, New York, NY, USA; 5 Department of Anthropology, 2546 Case Western Reserve University, Cleveland, OH, USA; 6 59097 SA MRC Unit on Risk & Resilience in Mental Disorders, Department of Psychiatry & Neuroscience Institute, University of Cape Town, Cape Town, South Africa; 7 Department of Medicine and Neuroscience Institute, University of Cape Town, Cape Town, South Africa; 8 Psychology Department, 192255 Rhodes University, Grahamstown, South Africa; 9 Department of Psychiatry and Mental Health, University of Cape Town, Cape Town, South Africa

**Keywords:** internalised stigma, ISMI, mental illness, schizophrenia, South Africa, translation, Xhosa

## Abstract

Internalised stigma is highly prevalent among people with mental illness. This is concerning because internalised stigma is often associated with negative consequences affecting individuals’ personal, familial, social, and overall wellbeing, employment opportunities and recovery. Currently, there is no psychometrically validated instrument to measure internalised stigma among Xhosa people in their home language. Our study aimed to translate the Internalised Stigma of Mental Illness (ISMI) scale into isiXhosa. Following WHO guidelines, the ISMI scale was translated using a five-stage translation design which included (i) forward-translation, (ii) back-translation, (iii) committee approach, (iv) quantitative piloting, and (v) qualitative piloting using cognitive interviews. The ISMI isiXhosa version (ISMI-X) underwent psychometric testing to establish utility, within-scale validity, convergent, divergent, and content validity (assessed using frequency of endorsements and cognitive interviewing) with *n* = 65 Xhosa people with schizophrenia. The resultant ISMI-X scale demonstrated good psychometric utility, internal consistency for the overall scale (α = .90) and most subscales (α > .70, except the Stigma Resistance subscale where α = .57), convergent validity between the ISMI Discrimination Experiences subscale and the Discrimination and Stigma (DISC) scale's Treated Unfairly subscale (isiXhosa version) (*r* = .34, *p* = .03) and divergent validity between the ISMI Stigma Resistance and DISC Treated Unfairly subscales (*r* = .13, *p* = .49). But more importantly the study provides valuable insights into strengths and limitations of the present translation design. Specifically, validation methods such as assessing frequency of endorsements of scale items and using cognitive interviewing to establish conceptual clarity and relevance of items may be useful in small piloting sample sizes.

## Introduction

Xhosa people (known as amaXhosa) are South African Bantu speakers and form part of the Nguni language-speaking ethnic groups in Southern Africa. They constitute the second largest population group (14.8%) in South Africa (The World Factbook, 2021). While most Xhosa people originate from the Eastern Cape Province (Buhrmann, 1977), today, many Xhosa people live in different areas of the country—which vary in levels of urbanisation. Historically, they form part of the population groups that were discriminated against during the apartheid era in South Africa. Xhosa people with schizophrenia are a small minority. Like many other Xhosa people, most contend with resource inequalities—including education and healthcare services—in addition to experiences of discrimination and stigmatisation related to mental illness (Botha et al., 2006; Mbanga et al., 2002).

Internalised/self-stigma is characterised by shame and the expectation of discrimination when a person discloses a condition that is vulnerable to stigma (Corrigan & Watson, 2002; Livingston & Boyd, 2010). Such internalised stigma can be conceptualised as the internalisation of negative attitudes, stereotypes, and perceptions of people who belong to a devalued social group (Corrigan & Watson, 2002). It is estimated that between 30%–50% of people with schizophrenia globally experience internalised stigma (Gerlinger et al., 2013). Internalised stigma is often associated with negative consequences affecting individuals’ personal, familial, social and overall wellbeing, employment opportunities and their recovery (Link & Phelan, 2001). A qualitative study found that Xhosa people with schizophrenia experience internalised stigma in the form of stereotypes, prejudice, and discrimination (Matshabane et al., 2020). Notwithstanding this work, the lack of instruments to quantitatively measure internalised stigma in this cultural group limits our understanding of this phenomenon. While there is some research on internalised stigma and mental illness among African people (Adewuya et al., 2011; Assefa et al., 2012) and among South African people specifically (Matshabane et al., 2020; Mbanga et al., 2002), to date no standardised instrument exists to measure stigma experiences in the isiXhosa language. This article therefore details the key learnings that arose while translating the Internalised Stigma of Mental Illness (ISMI) scale into isiXhosa (hereafter referred to as the ISMI-X) and piloting it for use among Xhosa-speaking people with schizophrenia.

## The measure

Globally, several measures have been developed to assess stigma experiences of people living with a mental illness. However, the ISMI scale was one of only two measures that met psychometric quality criteria in Stevelink et al.'s (2012) systematic review of 21 available internalised stigma scales. The original ISMI scale is a 29-item tool comprising 5 subscales: four internalised stigma experience subscales, including: Alienation (6 items), Stereotype Endorsement (7 items), Discrimination Experience (5 items) and Social Withdrawal (6 items); and one resilience towards stigma subscale, Stigma Resistance (Ritsher et al., 2003).

The ISMI scale has demonstrated high internal consistency across all subscales (α = .74–.92) (Boyd et al., 2014; Ersoy & Varan, 2007; Kira et al., 2011; Ritsher et al., 2003; Singh et al., 2016; Wong-Anuchit et al., 2016), except the Stigma Resistance subscale (Lysaker et al., 2006). The measure has shown acceptable convergent validity, comparing favourably with the Devaluation-Discrimination Scale and the Discrimination and Stigma Scale (DISC) (Brohan et al., 2013; Ritsher et al., 2003). With identical methodology, the ISMI has also demonstrated acceptable divergent validity with low correlations with scales measuring different constructs such as the Personal Empowerment Scale (Ritsher et al., 2003). The tool has been translated into different languages for use in varying cultures and health conditions in mostly European and North American contexts where psychometric properties suggest good transportability (Boyd et al., 2014). These findings indicate that the ISMI is a theoretically and psychometrically sound tool for quantifying internalised stigma experiences across a broad range of countries, languages, and cultures. A recent meta-analysis, however, has called for an increase in studies measuring internalised stigma in underrepresented populations, especially those from different geographical locations (Del Rosal et al., 2021).

The ISMI scale has been adapted for use in three African countries: a Yoruba version was developed in Nigeria (Adewuya et al., 2011); an Amharic version in Ethiopia (Assefa et al., 2012); and a Lugandan version in Uganda (Boyd et al., 2014). The ISMI Yoruba version demonstrated high internal consistency (α = >.84) and test-retest reliability (*r*_kk_ = .86) (Adewuya et al., 2011). The ISMI Amharic version also demonstrated high internal consistency (α = >.92), but validity was not reported (Assefa et al., 2012). No psychometric properties were reported for the Luganda version (Boyd et al., 2014). In the South African context, the ISMI English version has been used in two studies (Botha et al., 2006; Sorsdahl et al., 2012). Sorsdahl et al. (2012) investigated internalised stigma experiences among highly educated, predominantly White, English-speaking South African members of a mental health support group. Botha et al. (2006) used the ISMI English version in a study including Afrikaans (*n* = 77), English (*n* = 17) and a small number of isiXhosa-speaking South African people (*n* = 6) with severe mental illness. An important finding from Botha et al.'s study is that Xhosa people with schizophrenia reported a higher incidence of abuse experiences than the other two groups.

Based on this evidence, we sought to translate the ISMI into an isiXhosa-language version for use with South African isiXhosa-speaking people with schizophrenia. Drawing from Botha et al.'s (2006) findings about the prevalence and potential impact of abuse on internalised stigma experiences among South African Xhosa people, we included a sixth subscale measuring experiences of abuse (6 items obtained from Botha et al., 2006), resulting in a 35-item instrument.

## Methodology

This study was approved by the University of Cape Town, Health Sciences Research Ethics Committee (FHS204-2015). All participants provided informed consent for participation in this study.

### Translation design

Applying a mixed-methods approach, we followed a five-stage translation design in accordance with WHO guidelines (Sartorius & Janca, 1996) to develop as valid an isiXhosa translation of the ISMI scale as possible. The stages included: (i) *Forward-translation*, (ii) *Committee Approach*, (iii) *Back-translation*, (iv) *Quantitative Pilot*, and (v) *Qualitative Pilot* (Campbell & Young, 2016).

### Translation team

Our bilingual translation team included six home language isiXhosa-speaking health research project staff, including four who have had tertiary training at English-language institutions: two male psychiatric nurses who worked regularly with schizophrenia patients; a male and a female health research professionals who have extensive experience translating instruments from English to isiXhosa; a female retired psychiatric nurse and research coordinator with extensive clinical and research experience engaging with Xhosa people about their experiences of schizophrenia in isiXhosa; and the lead author of this paper who is trained in research psychology.

We selected these translators for two reasons. First, because isiXhosa is their home language, they were knowledgeable about the Xhosa culture. We avoided using translators who were only versed in the source and target languages with no understanding or experience of the culture and of conducting research with people who have a health condition. We anticipated that the use of strictly language competent translators could result in a translation that does not capture the ways in which Xhosa people who have a mental illness make sense of their condition. Second, we involved two general health professionals in addition to the psychiatric research nurses in an attempt to limit the potential bias of only including personnel who are trained in mental health and who may therefore be overly familiar with the conceptual domains of the scale. This strategy assisted us in enhancing the applicability of the scale for lay Xhosa people who have schizophrenia.

### Translation procedure

First, the tool was forward translated into isiXhosa by the two psychiatric nurses and two general health professionals. The translations were compiled in a table by the first author and discussed at a committee meeting with the translation team, including the first and senior authors, after which a version was finalised for piloting.

Second, the tool was quantitatively piloted with 65 isiXhosa-speaking people with schizophrenia as part of a battery of psychological measures administered in the Genomics of Schizophrenia in South African Xhosa people (SAX) project (Gulsuner et al., 2020). The purpose of piloting the instrument was to discern how speakers of the target language responded to the instrument and how they understood the individual items, which can reveal important information about how internalised stigma is understood in this cultural group, and in turn could help to revise the final tool. Of the 65 participants who completed the ISMI-X, 32 also completed the isiXhosa version of the DISC scale as a measure of convergent validity. The DISC is an internationally established measure of discrimination and stigma experiences of people with schizophrenia; the English version of the tool has been used as a measure of convergent validity with the ISMI in a UK study (Brohan et al., 2013). Patient recruitment, consent and administration of these measures were completed by trained psychiatric nurses at psychiatric hospitals in both the Eastern and Western Cape provinces in South Africa.

Third, the forward translation was back-translated into English by an additional psychiatric nurse who was not familiar with the original ISMI tool and had not been involved in the forward translation. There is some debate in the literature about the advantages and disadvantages of using healthcare professionals as back translators in a translation team, primarily because healthcare professionals may back-translate scales into clinical terminology which is different from patient understanding as they may not be able to separate their conceptual knowledge of the constructs measured in the scale from the back-translation they produce. To compensate for the potential limitation of using a psychiatric nurse for back-translation in our study, we included a cognitive interviewing component in our translation design, to explore how isiXhosa-speaking people with schizophrenia were understanding each item on the internalised stigma scale.

Fourth, using the method of cognitive interviews, the ISMI-X was qualitatively piloted by the first author on five isiXhosa-speaking schizophrenia inpatients. The aim of the cognitive interviews was to explore the conceptual equivalence of item wording in the ISMI-X scale. The benefit of using cognitive interviews is that they can allow for efficient identification of translated items that require further revision. Interviews were conducted after participants had completed the ISMI-X. The first author then asked participants to reflect on each ISMI-X item and answer two questions:

1. *What did the question make you think of?*
2. *Why did you respond in the way you did to this question?*

Fifth, the resultant quantitative and qualitative data as well as the back-translation were discussed in a second committee meeting with the translation team and all discrepancies were debated and resolved, resulting in a final ISMI isiXhosa version. The second committee meeting was important because it created an opportunity for the translation team to review all the evidence, deliberate on discrepancies and finalise a scale with the most accurate translations. The entire translation process was conducted over 9 months. While this is a lengthy period of time, we believe that following this robust translation process lessened the chances of developing a scale with inaccurate translations which may ultimately lack validity for the target cultural group. Furthermore, not conducting a thorough process may result in semantic differences which could lead to inaccurate results that could be interpreted as cultural differences in experiences or attitudes but in turn may be due to linguistic errors.

### Data analysis

Psychometric properties of the ISMI-X were investigated using measures of utility, reliability, and validity. Utility was assessed using completion rates and score distributions (Lamping et al., 2002). Internal reliability was calculated across the entire scale and subscales using Cronbach's alpha (Cronbach, 1951). Within-scale validity was established using correlations of the ISMI-X subscales with full scale scores using Spearman's Rho. Convergent validity was measured using the relationship between the ISMI-X subscales (24 items) and the DISC Treated Unfairly isiXhosa version subscale (22 items), based on the theoretical relationship between stigma and discriminatory behaviour (Brohan et al., 2013); the Treated Unfairly subscale can be used as a stand-alone measure of perceived stigma and discrimination (Brohan et al., 2013). For divergent validity we investigated the relationship between the ISMI-X Stigma Resistance subscale and DISC Treated Unfairly isiXhosa version subscale, as these two scales are theorised to measure different constructs (Brohan et al., 2013). Only participants who completed both the ISMI-X and the DISC isiXhosa version scales and had fewer than 3 items omitted in each scale were included in the convergent and divergent validity analyses (*n* = 32) where we compared scale scores using Spearman's Rho. Content validity was investigated quantitatively by examining the frequency of item endorsements for each ISMI-X subscale and qualitatively using results from the cognitive interviews. Data were managed in Microsoft Excel and analyses were conducted using SPSS (Version Q3 25.0). Item endorsements by 30% of participants or more were considered meaningful in accordance with results of the multinational review of ISMI scales (Boyd et al., 2014).

## Results

### Socio-demographic information

Our sample of 65 isiXhosa-speaking people with schizophrenia included 60 participants for the quantitative analysis and 5 participants for the qualitative analysis. Male participants were over-represented in the total sample (*n* = 57, 95%), with an age range of 20–53 years and a mean age of 30.3 years. Most participants had some secondary schooling (*n* = 45, 75%) and were at the time unemployed (*n* = 54, 90%).

### Utility

Of the 60 ISMI-X questionnaires administered, 47 (78.33%) were completed in full, 9 (15%) had one item omitted, 0 (0%) had two items omitted, while only 4 (4.66%) had three or more items omitted. These figures suggest that the tool was fairly easy to read, understand and complete.

### Internal consistency

Consistency for the ISMI-X total scale and subscales was high and congruent with the results reported for Boyd's original ISMI English version (Ritsher et al., 2003). Results are summarised in Table 1.

**Table 1.** Internal consistency of ISMI original English and isiXhosa language versions.

	*Original English* Cronbach's α	ISMI-X Cronbach's α	[95% CI]
ISMI full scale	.91	.90	[0.76, 0.91]
Alienation subscale (A) – 6 items	.79	.73	[0.60, 0.83]
Stereotype Endorsement subscale (SE) – 7 items	.72	.82	[0.74, 0.88]
Discrimination Experience subscale (DE) – 5 items	.75	.78	[0.67, 0.86]
Social Withdrawal subscale (SW) – 6 items	.80	.86	[0.79, 0.91]
Stigma Resistance subscale (SR) – 5 items	.58	.57	[0.36, 0.73]
Abuse subscale (AB) – 6 items	-	.78	[0.68, 0.86]

### Validity

Moderate positive correlations (*r* = .31–.63) were found between the ISMI-X total and subscale scores, suggesting acceptable within-scale validity. Results are summarised in Table 2. With respect to convergent validity neither the ISMI-X total score nor the Alienation, Stereotype Endorsement, Social Withdrawal or Stigma Resistance subscales showed any significant association with the DISC Treated Unfairly subscale. Only the ISMI-X Discrimination Experience subscale and the DISC Treated Unfairly subscale demonstrated a moderate positive correlation (*r* = .34, *p* = .03). In terms of divergent validity, as hypothesised, the ISMI-X Stigma Resistance subscale did not show a significant association with the DISC Treated Unfairly subscale (*r* = .13, *p* = .49). Results of the psychometrics are summarised in Table 3.

**Table 2.** Within-scale correlations for the ISMI-X using Spearman's Rho (*n* = 56).

	ISMI-X	A	SE	DE	SW	SR	AB
ISMI-X		0.78*	0.79**	0.71**	0.85*	0.42*	0.64**
A		1.00	0.49*	0.46	0.51	0.31*	0.48**
SE			1.00	0.31*	0.60**	0.51	0.50
DE				1.00	0.53**	0.18	0.37**
SW					1.00	0.29	0.63**
SR						1.00	0.18

*Note*. A = Alienation, SE = Stereotype Endorsement, DE = Discrimination Experience, SW = Social Withdrawal, SR = Stigma Resistance, AB = Abuse).

*p < .05. **p < .01.

**Table 3.** Correlations between ISMIS-X and DISC Treated Unfairly subscale.

	DISC Treated Unfairly Spearman's Rho (*n* = 32)	
ISMI-X (excluding Stigma Resistance)	*r* = .10	*p* = .60
Alienation Subscale (A)	*r* = .23	*p* = .10
Stereotype endorsement Subscale (SE)	*r* = −.08	*p* = .33
Discrimination experience Subscale (DE)	*r* = .34*	*p* = .03
Social withdrawal Subscale (SW)	*r* = .08	*p* = .33
Stigma resistance Subscale (SR)	*r* = .13	*p* = .24

*p < .05.

**Figure 1.**
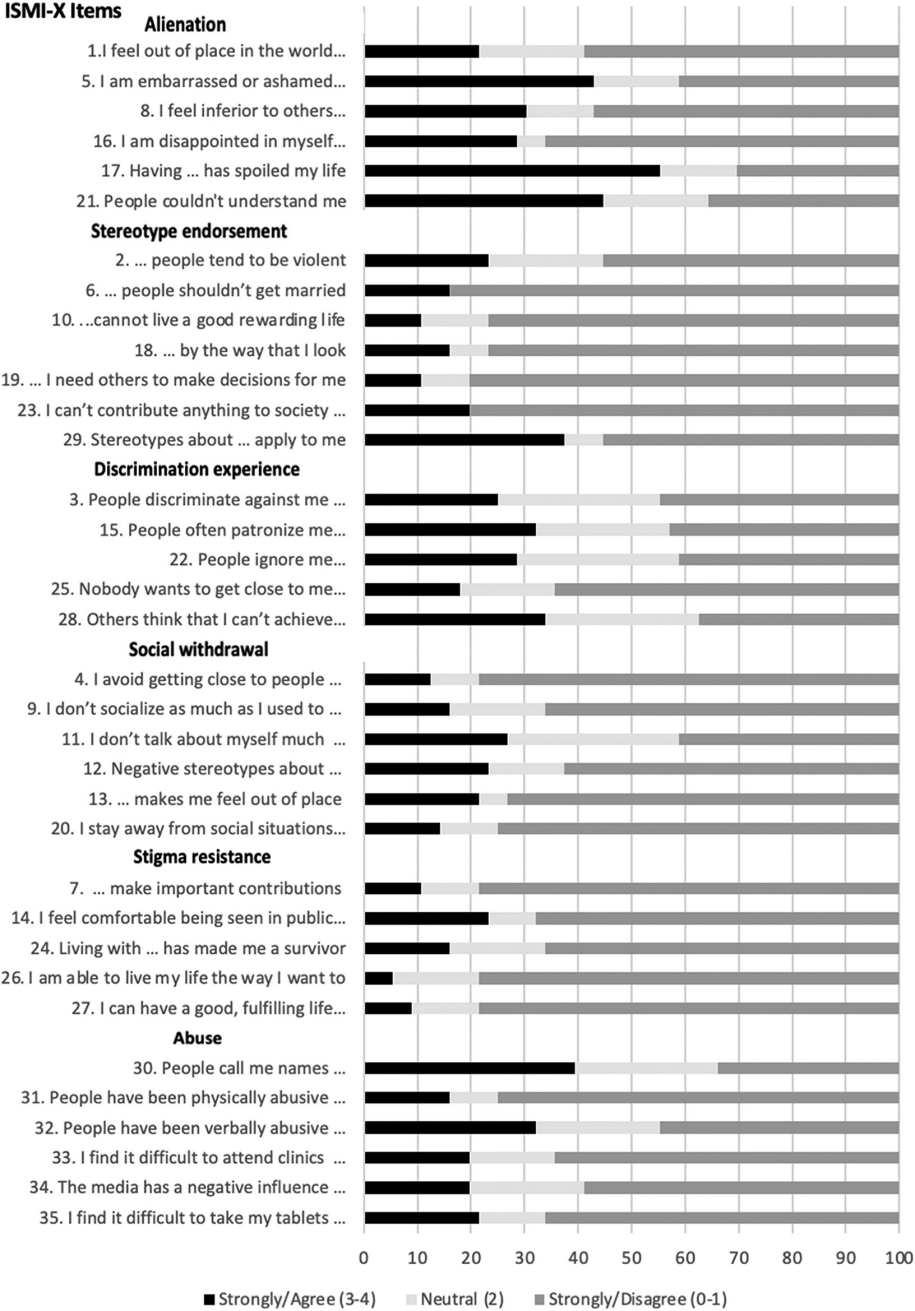
ISMI-X Frequency endorsements.

Content validity was highest for the Alienation and Abuse subscales. The most frequently endorsed items in the Alienation scale were items 17: “*Having a mental illness has spoiled my life*”, 21: “*People without mental illness could not possibly understand me*”, and 5: “*I am embarrassed or ashamed that I have a mental illness*”. These items were followed by items in the Abuse subscale, 30: “*People call me names because I have a mental illness*” and 32: “*People have been verbally abusive to me because I have a mental illness*”. The least endorsed subscales were the Stereotype Endorsement, Discrimination Experience, Social Withdrawal and Stigma Resistance subscales with most items being minimally endorsed. In the Stereotype Endorsement subscale, participants moderately endorsed item 29: “*Stereotypes about the mentally ill apply to me*”, but did not endorse items referring to specific examples of stereotypes. In the Discrimination Experience subscale, the items which were moderately endorsed are 28: “*Others think I can't achieve much because I have a mental illness*” followed by 15: “*People often patronise me, or treat me like a child, just because I have a mental illness*”. Frequency of endorsements are summarised in Figure 1, reasons for less endorsement of specific items by participants in the cognitive interviews are summarised in Table 4, and discrepancies found in back-translations are summarised in Table 5.

**Table 4.** Examples of translation issues identified in cognitive interviews.

Translation issues	Item examples	Findings
isiXhosa word does not convey intended construct	Item 23: “I can't contribute anything to society because I have a mental illness” which was translated / “*Andikwazi ukuba negalelo ekuhlaleni nakweyiphi na into kuba ndiphazamisekile engqondweni*” Item 29: “Stereotypes about the mentally ill apply to me” /“*Iingcamango ngabantu abagula ngengqondo ziquka nam*”	The word “society” was understood as “community” by all participants. All participants understood the item to refer to giving back (i.e., financially or through physical labour) to their community. Participants struggled to understand words like “stereotype”. Two participants understood it as “thoughts” and the others did not understand or relate to the item at all.
English idiom in item is difficult to understand	Item 1: “I feel out of place in the world because I have a mental illness”/ “*Ndiziva ndingamkelekanga emhlabeni kuba ndiphazamisekile engqonweni*” Item 24: “Living with mental illness has made me a tough survivor” / “*Ukuphila ndinophazamiseko engqondweni kundenze ndaphumelela kwimeko ezinzima*”	All participants did not understand the idiom “out of place in the world” however two related it to feeling unappreciated or feeling like they do not belong. All participants had difficulty understanding the idiom “tough survivor”. Two participants related “tough survivor” to their ability to survive but did not reflect on the euphemism. Another related it to remaining positive in all situations. The other participants did not understand the item at all.
Identifying with part of the item but not its entirety	Item 9: “I don't socialise as much as I used to because my mental illness might make me look or behave weird” / “*Andisakonwabeli kakhulu ukuhlala nabanye abantu njengoko ndandiqhele ukwenza kuba isigulo sam sengqondo sindenza ndibonakale kwaye ndenze izinto ezingaqhelekanga*” Item 4: “I avoid getting close to people who don't have a mental illness to avoid rejection” / “*Ndiyazikhwebula ukuba kufutshane kubantu abangaphazamisekanga engqondweni ukuze ndingaziva ndingamkelekanga*”	For both items 9 and 4, all participants reported difficulty in answering these items explaining that they relate to the first part of the question “I don't socialise” and “I avoid getting close to people”, in a general sense and not necessarily solely avoid people who are not mentally ill. Their avoidance of people was also not because of their fear of looking or behaving in an inappropriate manner.

**Table 5.** Examples of language differences identified in the back-translation.

	Original items	ISMI-X items	Back-translated items
1	I feel out of place in the world because I have a mental illness	*Ndiziva ndingamkelekanga emhlabeni kuba ndiphazamisekile engqonweni*	Due to my **mental instability**, I **don't feel welcome in the world**.
5	I am embarrassed or ashamed that I have a mental illness	*Ndiziva ndinodano okanye ndinentloni kuba ndiphazamisekile engqondweni*	I feel embarrassed or ashamed because I **suffer** from mental illness.
16	I am disappointed in myself for having a mental illness	*Ndiziva ndinodano ngesiqu sam kuba ndiphazamisekile engqondweni*	I feel **ashamed** of myself as a person due to having a mental illness.
29	Stereotypes about the mentally ill apply to me	*Iingcamango ngabantu abagula ngengqondo ziquka nam*	I am included in the **opinions** society has on people with a mental illness.

## Discussion

When needing to quantify constructs like internalised stigma, researchers are often faced with the choice of translating and adapting established measures or developing new context-specific scales. This choice is typically dictated by available time and financial resources. In studies that choose to translate established tools, a pragmatic challenge is generating a large enough sample size during piloting of the translated language version to run the necessary psychometric analyses to establish validity.

Our study found specific challenges in establishing content validity of stigma constructs from English into isiXhosa, suggesting difficulty in transportability of these constructs across the two languages. These challenges included cultural differences in conceptualisation of mental illness and internalised stigma across the two languages, as well as limited availability of direct translations for key English terms in isiXhosa that also resulted in difficulties denoting intensity of particular terms. A key finding from our study is the value that methods such as frequency of endorsement of scale items and cognitive interviewing add in evaluating the validity of translated measures using a small sample size. The following examples are illustrative.

Across the ISMI-X scale, items from the Alienation and Abuse subscales were the most frequently endorsed by our participants. In particular, items referring to feelings of shame, embarrassment, and inferiority about having a severe mental illness like schizophrenia appeared the most relevant and meaningful for our participants, in addition to how the illness had ruined participants’ lives and created a barrier between them and others without a mental illness. Participants also endorsed items relating to being called names or experiencing abuse from others because of their mental illness. These results are similar to findings from previous internalised stigma studies, which found the Alienation subscale to be most frequently endorsed (Brohan et al., 2010; Sorsdahl et al., 2012) and the Abuse items 30 and 32 to be particularly relevant for Xhosa people with schizophrenia (Botha et al., 2006; Matshabane et al., 2020). One possible reason for the increased reports of abuse within this group may be due to the contextual environments in which they reside. Violence is a common phenomenon in the South African township communities—where most of our participants come from. Living in a community with high levels of violence may place people with mental illness at an increased risk of experiencing abuse. Cognitive interviews further substantiated this finding with participants describing experiences of people calling them names and not treating them well, which may be a contributing factor to the negative feelings about themselves.

Within the Stereotype Endorsement subscale, “Stereotypes about having a mental illness apply to me” (item 29) was the most frequently endorsed item. Interestingly, the remaining items in this subscale, describing practical examples of stereotypes the public may hold about people with schizophrenia such as that they are violent, should not get married, and cannot live a productive life, were not frequently endorsed by the participants in our study. In previous studies the Stereotype Endorsement subscale has often been the least endorsed subscale (Boyd et al., 2014; Lysaker et al., 2007; Sorsdahl et al., 2012). In our study, cognitive interviews indicated that this was because participants did not identify these items as beliefs others may hold of them, which suggests that in the Xhosa culture, there may be other kinds of stereotypes related to mental illness. For instance, Matshabane et al. (2020) found that Xhosa people in that study often associated people who have symptoms of schizophrenia with being “dirty”. The stereotype of “dirtiness” being associated as an experience of people with mental health challenges—and in this case schizophrenia—has been reported to be commonly held by the South African general public (Hugo et al., 2003), traditional healers (Sorsdahl et al., 2010) and by caregivers of Xhosa people with schizophrenia (Mbanga et al., 2002). This concept is not captured in the ISMI and may be one notable way in which Xhosa people with mental illness self-stigmatise themselves. Adding a related item in the Stereotype Endorsement subscale such as “People with mental illness tend to be dirty” may therefore be relevant for this group.

In this study, a combination of low item endorsement and qualitative feedback during cognitive interviewing was particularly helpful in highlighting problematic scale items. For example, the item containing an English idiom, “*I feel out of place in the world because I have a mental illness*“ (item 1), was translated into “*Ndiziva ndingamkelekanga ehlabathini kuba ndiphazamisekile ngokwasengqondweni*”, which back-translates to “*I feel unwelcome in the world because I have a mental illness*.” This item from the Alienation subscale did not resonate as a particularly meaningful stigma experience for our participants. Cognitive interviews suggested that participants understood this item as relating to feeling like one does not belong or being unappreciated. Responses suggest that participants do feel like they belong in the world. This may not be surprising given that many African cultures, including the Xhosa culture, emphasise communal values (Wiredu & Gyekye, 1992) and a sense of belonging. Although not all African people hold this worldview, a majority of cultural groups in sub-Saharan Africa do have a firm belief that the individual is not considered in isolation, but rather in a relational manner with the greater community, and therefore they have a sense of belonging (Nwoye, 2015).

Another example from the Stigma Resistance subscale is item 21, “*Living with a mental illness has made me a tough survivor*”, which was translated to “*Ukuphila ndinophazamiseko lwengqondo kundenze ndaphumelela kwimeko ezinzima*” and back translates to “*Living with a mental disorder has seen me get out of difficult situations*”. The euphemism “tough survivor” was difficult for the translators to translate into isiXhosa, and they spent a significant amount of time debating how to phrase this item in the meetings. Ultimately, the final translation—as evident from the back translation—does not adequately capture the intensity described in the English language. Additionally, the cognitive interviews revealed that all participants did not understand this item as originally intended. Even though two participants related it to the ability to survive difficult situations, they did not consider it in relation to their identity (i.e., referring to themselves as a “tough survivor”).

Item 7, “*People with mental illness make important contributions to their society*“ (from the Stigma Resistance subscale) is another useful example. There is no direct isiXhosa translation for “society” and the closest approximation, “ekuhlaleni”, back translates to “community”. In this context, community refers to people living in close proximity and considering that society refers to a larger system of social relationships, there is a distinct difference in the intended contextual meaning. Feedback during cognitive interviews indicates that participants, coming from a background of minimal financial resources (most participants were unemployed) and having a condition that compromises functional ability, were unsure of the kind of contribution this item could be referring to and how it would be perceived as valuable within their immediate community. Additionally, in the Xhosa culture, meaningful contributions to the community often include financial contributions, for instance in cases where family members need money to support another family member's health or educational costs. It could also refer to physical contributions—such as using one's physical strength to “dig a grave” ahead of the funeral of a family or community member (Matshabane et al., 2020). Being able to engage in these actions and contribute in these ways has valuable social meanings in this cultural group.

Examples from our research highlight how frequency of endorsements used in combination with cognitive interviews provide valuable information about the content validity of scale items in a relatively small sample size. In our study, these data highlighted some important challenges related to the transportability of stigma constructs from English to the isiXhosa language. The differences may be illustrative of the influence of cultural understandings on the construct of mental illness and stigma. We know that language and culture are intertwined and that they play an integral role in the construction of meaning (Swartz, 1998). This suggests that thoughts, feelings and emotions are governed by the cultural background, vocabulary and sentence structure available in a language (Swartz, 1998). With that knowledge and the evidence from our study, it is difficult to conclude that the ISMI-X can be used cross-culturally in comparison to data emanating from Western cultural contexts. This is because, whilst studies in the United States, Europe and the UK have consistently demonstrated strong psychometric properties of the ISMI scale (Boyd et al., 2014), suggesting good transportability in these contexts, there have been cross-cultural challenges to adapting the scale for the African context (Adewuya et al., 2011). Similarly, in Latin American countries the ISMI has not met psychometric properties and researchers Caqueo-Urízar et al. (2019) have therefore advised for the full scale not to be used in Latin American countries but have rather recommended a shorter version of the scale containing 12 items that are most relevant for Latin American cultural groups. This may not be too surprising given that while languages may differ, Western concepts of psychiatric disorders appear quite consistent in European cultural contexts (Boyd et al., 2014). In contrast, different conceptual models of mental illness may be present in African and other indigenous cultural groups. In this case, that means more caution should be taken when interpreting ISMI scores across African cultural groups (Campbell, Matshabane, et al., 2021; Campbell, Sibeko, et al., 2017; Naanyu, 2010; Sorsdahl et al., 2010). A particular strength of this research, though, is that it provides valuable insights into more context, culture, and language-specific expressions of internalised stigma in an African setting.

In our study, another reason for the low frequency of item endorsement in the ISMI-X may be a less marked association between discrimination due to mental illness and internalised stigma in this cultural group. It is important to consider that Xhosa people have been and continue to be subjected to structural discrimination as a group, having contended with a history of systematic discrimination based on race. Arguably, structural and disease stigma places Xhosa people in a position of experiencing “intersectionality stigma” (Bowleg, 2012). Intersectionality stigma theory emphasises that some groups have multiple stigmatised identities (such as race, gender, and class) and the consequences of membership in multiple stigmatised groups requires us to consider the combined effects of the stigmatising factors, rather than each one exclusively (Rice et al., 2018). For this cultural group, because they are contending with stigma not only because they have a mental illness, but also because they are Black men, mostly unemployed, have had unequal access to education and healthcare and reside in low-income township communities—it may be important to investigate those factors concurrently. This is what is described through the double disadvantage theory (Denise, 2014; Dowd & Bengtson, 1978), where there is an accumulation of disadvantage based on an increased number of stigmatised identities (Oexle & Corrigan, 2018).

Finally, it is possible that the lack of endorsement of some of the ISMI-X items may be evidence of the acceptance of the everyday discrimination that Xhosa people have historically experienced and continue to experience, including racial and socioeconomic discrimination, suggesting that having this disease label may not uniquely impact their discrimination experiences. This historically deep-rooted type of structural stigma and its consequences may supersede experiences of internalised stigma linked to disease for this cultural group. Relatedly, the concept of “stigma competence” (Balsam & D'Augelli, 2006; Isacco et al., 2012) may be useful. This is because, it may be, that the skills our participants have to navigate different forms of stigma, may have an unanticipated effect for raising their overall capacity to deal with disease‐stigma (American Psychological Association, 2015), hence the limited reports of perceived disease-related discrimination found in this study. We would emphasise that the lack of reports of discrimination does not necessarily indicate a lack of such discrimination, but rather may point to under-reporting of disease stigma related to an intersectionality with structural stigma.

## Strengths and limitations

A limitation of the ISMI-X is that it provides a broad description of the internalised stigma experiences of Xhosa people with schizophrenia which cannot be taken as a proxy for Xhosa people with other mental illnesses. The limited number of items that were frequently endorsed in this scale may suggest that further modifications would be useful for improving its relevance for this cultural group. Another limitation of this study is that the sample size was not powered for factor analysis. Furthermore, we also note the over-representation of males in our sample (95%) and the relatively young age of this group (average: 30.3 years). Although the male predominance is representative of the sex difference in the larger genomics study from which this sample was drawn (Gulsuner et al., 2020) and in other genomic studies which enrolled Xhosa people with schizophrenia (Campbell et al., 2017; Luckhoff et al., 2014), the resultant data may not adequately represent the views of older Xhosa people, as well as Xhosa women with schizophrenia.

Despite these limitations, there are a number of important strengths. First, the ISMI-X translation process provided valuable insights into how item endorsement and cognitive interviews can be used to consider the validity of a translated measure without large sample sizes. Second, this work applied a five-stage translation design. Third, the ISMI-X translation process was led by the first author, who is a home-language isiXhosa-speaker and is trained in psychology, psychiatry, and ethics. This is a strength in that the researcher has an in-depth understanding of the culture, language, and psychological constructs. Previous studies which have conducted translations of instruments from English to isiXhosa have been led by individuals who are not first-language speakers of the language or who do not share a cultural background with the target group. Finally, most studies that have translated scales from English to isiXhosa have only applied some but not all of the five steps followed in this study.

In conclusion, this article makes a unique contribution to the literature by documenting the key findings that arose while translating the ISMI scale into isiXhosa. The ISMI-X scale developed through this study is available for other researchers to use and develop further.

## Supplemental Material

sj-docx-1-tps-10.1177_13634615231168461 - Supplemental material for Lessons learned from the translation of the Internalised Stigma of Mental Illness (ISMI) scale into isiXhosa for use with South African Xhosa people with schizophrenia Supplemental material, sj-docx-1-tps-10.1177_13634615231168461 for Lessons learned from the translation of the Internalised Stigma of Mental Illness (ISMI) scale into isiXhosa for use with South African Xhosa people with schizophrenia by Olivia P. Matshabane, Paul S. Appelbaum, Marlyn C. Faure, Patricia A. Marshall, Dan J. Stein, Jantina de Vries, and Megan M. Campbell in Transcultural Psychiatry
