# Supplementary material for: Lessons learned from the translation of the Internalised Stigma of Mental Illness (ISMI) scale into isiXhosa for use with South African Xhosa people with schizophrenia
Source: Transcult Psychiatry. 2023 Jun 18;62(1):33–44. doi: 10.1177/13634615231168461 (PMC12089671; doi:10.1177/13634615231168461)
Supplement: sj-docx-1-tps-10.1177_13634615231168461 - Supplemental material for Lessons learned from the translation of the Internalised Stigma of Mental Illness (ISMI) scale into isiXhosa for use with South African Xhosa people with schizophrenia [file sj-docx-1-tps-10.1177_13634615231168461.docx]

**Appendix A**

Table 1: *Internal consistency of ISMI original English and isiXhosa language versions*

|  | **Original English**  Cronbach’s α | **ISMI-X**  Cronbach’s α (CI: 95%) |
| --- | --- | --- |
| ISMI Full Scale | 0.91 | 0.90 (CI:0.76-0.910) |
| Alienation Subscale (A) - 6 items | 0.79 | 0.73 (CI:0.596-0.827) |
| Stereotype Endorsement Subscale (SE) - 7 items | 0.72 | 0.82 (CI:0.737-0.884) |
| Discrimination experience Subscale (DE) - 5 items | 0.75 | 0.78 (CI:0.669-0.859) |
| Social withdrawal Subscale (SW) - 6 items | 0.80 | 0.86 (CI:0.788-0.907) |
| Stigma resistance Subscale (SR) - 5 items | 0.58 | 0.57 (CI:0.362-0.732) |
| Abuse Subscale (AB) - 6 items | - | 0.78 (CI:0.675-0.860) |

Table 2: *Within-scale correlations for the ISMI-X using Spearman’s Rho (n= 56)*

|  | **ISMI-X** | **A** | **SE** | **DE** | **SW** | **SR** | **AB** |
| --- | --- | --- | --- | --- | --- | --- | --- |
| **ISMI-X** | 1.000 | 0.78^*^ | 0.79^**^ | 0.71** | 0.85^*^ | 0.42^*^ | 0.64** |
| **A** |  | 1.000 | 0.49^*^ | 0.46 | 0.51 | 0.31^*^ | 0.48^**^ |
| **SE** |  |  | 1.000 | 0.31^*^ | 0.60^**^ | 0.51 | 0.50 |
| **DE** |  |  |  | 1.000 | 0.53^**^ | 0.18 | 0.37^**^ |
| **SW** |  |  |  |  | 1.00 | 0.29 | 0.63^**^ |
| **SR** |  |  |  |  |  | 1.000 | 0.18 |

*(A= Alienation, SE= Stereotype Endorsement, DE= Discrimination Experience, SW= Social Withdrawal, SR= Stigma Resistance, AB= Abuse).*

*p < 0.05*; p < 0.01***

Table 3: *Correlations between ISMIS-X and DISC Treated Unfairly subscale*

|  | DISC Treated Unfairly  Spearman’s Rho (n= 32) |
| --- | --- |
| ISMI-X (excluding Stigma Resistance) | *r*= 0.10, p= 0.60 |
| Alienation Subscale (A) | *r*= 0.23, p= 0.10 |
| Stereotype Endorsement Subscale (SE) | *r*= - 0.08, p= 0.33 |
| Discrimination experience Subscale (DE) | *r*= 0.34*, p= 0.03 |
| Social withdrawal Subscale (SW) | *r*= 0.08, p= 0.33 |
| Stigma resistance Subscale (SR) | *r*= 0.13, p= 0.24 |

*p < 0.05*; p < 0.01***

Table 4: *ISMI-X Frequency endorsements*

Table 5: *Examples of translation issues identified in cognitive interviews*

| **Translation issues** | **Item examples** | **Findings** |
| --- | --- | --- |
| Xhosa word does not convey intended construct | Item 23: “I can’t contribute anything to society because I have a mental illness” which was translated /  “*Andikwazi ukuba negalelo ekuhlaleni nakweyiphi na into kuba ndiphazamisekile engqondweni”*  Item 29: “Stereotypes about the mentally ill apply to me” / “*Iingcamango ngabantu abagula ngengqondo ziquka nam”* | The word ‘society’ was understood as ‘community’ by all participants. All participants understood the item to refer to giving back (i.e. financially or through physical labour) to their community.  Participants struggled to understand words like ‘stereotype’. Two participants understood it as ‘thoughts’ and the others did not understand or relate to the item at all. |
| English idiom in item is difficult to understand | Item 1: “I feel out of place in the world because I have a mental illness”/  *“Ndiziva ndingamkelekanga emhlabeni kuba ndiphazamisekile engqonweni”*  Item 24: “Living with mental illness has made me a tough survivor” /  “*Ukuphila ndinophazamiseko engqondweni kundenze ndaphumelela kwimeko ezinzima”* | All participants did not understand the idiom “out of place in the world” however two related it to feeling unappreciated or feeling like they do not belong.  All participants had difficulty understanding the idiom ‘tough survivor’. Two participants related ‘tough survivor’ to their ability to survive but did not reflect on the euphemism. Another related it to remaining positive in all situations. The other participants did not understand the item at all. |
| Identifying with part of the item but not its entirety | Item 9: “I don’t socialise as much as I used to because my mental illness might make me look or behave weird” / “*Andisakonwabeli kakhulu ukuhlala nabanye abantu njengoko ndandiqhele ukwenza kuba isigulo sam sengqondo sindenza ndibonakale kwaye ndenze izinto ezingaqhelekanga”*.  Item 4: “I avoid getting close to people who don’t have a mental illness to avoid rejection” /  “*Ndiyazikhwebula ukuba kufutshane kubantu abangaphazamisekanga engqondweni ukuze ndingaziva ndingamkelekanga”*. | For both items 9 and 4, all participants reported difficulty in answering these items explaining that they relate to the first part of the question ‘I don’t socialise’ and ‘I avoid getting close to people’, in a general sense and not necessarily solely avoid people who are not mentally ill. Their avoidance of people was also not because of their fear of looking or behaving in an inappropriate manner. |

Table 6: *Examples of language differences identified in the back-translation*

|  | Original items | ISMI-X items | Back-translated items |
| --- | --- | --- | --- |

| 1 | I feel out of place in the world because I have a mental illness | *Ndiziva ndingamkelekanga emhlabeni kuba ndiphazamisekile engqonweni* | Due to my mental instability, I don't feel welcome in the world. |
| --- | --- | --- | --- |
| 5 | I am embarrassed or ashamed that I have a mental illness | *Ndiziva ndinodano okanye ndinentloni kuba ndiphazamisekile engqondweni* | I feel embarrassed or ashamed because I suffer from mental illness. |
| 16 | I am disappointed in myself for having a mental illness | *Ndiziva ndinodano ngesiqu sam kuba ndiphazamisekile engqondweni* | I feel ashamed of myself as a person due to having a mental illness. |
| 29 | Stereotypes about the mentally ill apply to me | *Iingcamango ngabantu abagula ngengqondo ziquka nam* | I am included in the opinion’s society has on people with a mental illness. |

Appendix B

|  | **Internalised Stigma of Mental Illness isiXhosa Version (ISMI-X)** | | | | |  |
| --- | --- | --- | --- | --- | --- | --- |
|  | **Sex: Isini** | | **M / F** | | | |
|  | **Age: Iminyaka yakho** | |  | | | |
|  | **Highest level of education: Ibanga lemfundo eliphezulu** | | | | | |
|  | **Where do you live? Uhlala phi** | |  | | | |
|  | | | | | | |
|  | | 1 | 2 | 3 | 4 | |
|  |  | Strongly disagree/ *Andivumi kakhulu* | Disagree/ *Andivumi* | Agree/ *Ndiyavuma* | Strongly agree/ *Ndiyavuma kakhulu* | |
| ***Alienation*** | | | | | | |
| 1 | I feel out of place in the world because I have a mental illness *Ndiziva ndingamkelekanga ehlabathini kuba ndiphazamiseke ngokwasengqondweni* |  |  |  |  | |
| 2 | I am embarrassed or ashamed that I have a mental illness  *Ndiziva ndiphoxekile okanye ndinentloni kuba ndiphazamiseke ngokwasengqondweni* |  |  |  |  | |
| 3 | I feel inferior to others who  don’t have a mental illness  *Ndiziva ndizeya kubantu abangaphazamisekanga engqondweni* |  |  |  |  | |
| 4 | I am disappointed in myself for having a mental illness  *Ndiziva ndinodano ngesiqu sam kuba ndiphazamiseke ngokwasengqondweni* |  |  |  |  | |

| 5 | Having a mental illness has spoiled my life  *Ukuba nesigulo sengqondo kubuphazamisile ubomi bam* |  |  |  |  |
| --- | --- | --- | --- | --- | --- |
| 6 | People without mental illness could not possibly understand me  *Abantu abangaphazamisekanga engqondweni ngekhe bayiqonde imeko yam* |  |  |  |  |
| ***Stereotype Endorsement*** | | | | | |
| 7 | Mentally ill people tend to be violent  *Abantu abaphazamisekileyo engqondweni bakholisa ukuba ndlongo-ndlongo* |  |  |  |  |
| 8 | Mentally ill people shouldn’t get  married  *Abantu abaphazamisekileyo engqondweni abafanelanga ukutshata* |  |  |  |  |
| 9 | People with a mental illness cannot live a good, rewarding life  *Abantu abaphazamisekileyo engqondweni abanakho ukuphila ubomi obulungileyo nobunembuyekezo* |  |  |  |  |
| 10 | People can tell that I have a mental illness by the way I look  *Abantu bangatsho ukuba ndiphazamisekile engqondweni ngendlela endibonakala ngayo* |  |  |  |  |
| 11 | Because I have a mental illness, I need others to make most decisions for me  *Kuba ndiphazamisekile engqondweni ndifuna abanye abantu bandithathele uninzi lwezigqibo* |  |  |  |  |

| 12 | I can’t contribute anything to society because I have a mental illness  *Andikwazi kuba nagalelo ekuhlaleni*  *nakweyiphi na into kuba ndiphazamisekile engqondweni* |  |  |  |  |
| --- | --- | --- | --- | --- | --- |
| 13 | Stereotypes about the mentally ill apply to me  *Iingcamango ezingezizo zabantu malunga nesigulo sengqondo nam ziyandichaphazela* |  |  |  |  |
| ***Discrimination Experience*** | | | | | |
| 14 | People discriminate against me because I have a mental illness *Abantu bayandicalu-*  *calula kuba ndiphazamisekile engqondweni* |  |  |  |  |
| 15 | People often patronize me, or treat me like a child, just because I have a mental illness  *Abantu bakholisa ukundiphatha okomntwana kuba ndiphazamisekile engqondweni* |  |  |  |  |
| 16 | People ignore me or take me less seriously  just because I have a mental illness  *Abantu baye bangandihoyi okanye bangandithatheli ngqalelo kuba ndiphazamisekile engqondweni* |  |  |  |  |
| 17 | Nobody would be interested in getting close to me because I have a mental illness  *Akukho mntu uyakuba nomdla wokusondela kum kuba ndiphazamisekile engqondweni* |  |  |  |  |

| 18 | Others think that I can’t achieve much in life because I have a mental illness  *Abanye abantu bacinga ukuba andinakuze ndiphumelele kangako ebomini kuba ndiphazamisekile engqondweni* |  |  |  |  |
| --- | --- | --- | --- | --- | --- |
| ***Social Withdrawal*** | | | | | |
| 19 | I avoid getting close to people who don’thave a mental illness to avoid rejection  *Ndiyazikhwebula ukuba kufutshane kubantu abangaphazamisekanga engqondweni ukuze ndingaziva ndingamkelekanga* |  |  |  |  |
| 20 | I don’t socialize as much as I used to because my mental illness might make me look orbehave weird *Andisakonwabeli kakhulu ukuhlala nabanye abantu njengoko ndandiqhele ukwenza kuba isigulo sam sengqondo sindenza ndibonakale kwaye ndenze izinto ezingaqhelekanga* |  |  |  |  |
| 21 | I don’t talk about myself much because I don’t want to burden others with my mental illness  *Andithethi kakhulu ngam kuba andifuni ukwenzela abanye abantu uxanduva ngesigulo sam sokuphazamiseka engqondweni* |  |  |  |  |
| 22 | Negative stereotypes about mental illness keep me isolated from the  ‘normal’ World  *Ingcamango ezimbi zithethwa ngophazamiseko lwengqondo zindenza ndiziguzule kwizinto eziqhubekayo ehlabathini 'eliqhelekileyo'* |  |  |  |  |

| 23 | Being around people who don’t have a mental illness makes me feel out of place or inadequate  *Ukuba phakathi kwabantu abangaphazamisekanga engqondweni kundenza ndizive ingathi ndilahlekile okanye ndimncinci* |  |  |  |  |
| --- | --- | --- | --- | --- | --- |
| 24 | I stay away from social situations in order to protect my family or friends from embarrassment  *Ndihlalela kude le kwindawo zolonwabo ukuze ndikhusele usapho lwam kunye nabahlobo bam kuhlazo* |  |  |  |  |
| ***Stigma Resistance*** | | | | | |
| 25 | People with mental illness make important contributions to society *Abantu abaphazamisekileyo engqondweni banegalelo elibalulekileyo ekuhlaleni* |  |  |  |  |
| 26 | I feel comfortable being seen in public with an obviously mentally ill person  *Ndiziva ndikhululekile esidlangalaleni nomntu ocacileyo ukuba uphazamisekile engqondweni* |  |  |  |  |
| 27 | Living with mental illness has made me a tough survivor *Ukuphila ndinophazamiseko lwengqondo kundenze ndaphumelela kwimeko ezinzima* |  |  |  |  |
| 28 | In general, I am able to live life the way I want to *Ngokuqhelekileyo, ndiyakwazi ukuphila ubomi ngendlela endifuna ngayo* |  |  |  |  |

| 29 | I can have a good, fulfilling life, despite my mental illness  *Ndingaba nobomi obuhle obanelisayo nangona ndiphazamisekile engqondweni* |  |  |  |  |
| --- | --- | --- | --- | --- | --- |
| ***Experiences of Abuse*** | | | | | |
| 30 | People call me names because I have a mental illness  *Abantu bandibiza ngamagama kuba ndiphazamisekile engqondweni* |  |  |  |  |
| 31 | People have been physically abusive towards me because I have a mental illness  *Abantu bebendihlukumeza ngokwasemzimbeni kuba ndiphazamisekile engqondweni* |  |  |  |  |
| 32 | People have been verbally abusive towards me because I have a mental illness  *Abantu bebethetha rhabaxa ngakum, kuba ndiphazamisekile ngokwase ngqondweni* |  |  |  |  |
| 33 | I find it difficult to attend clinic appointments because people will know that I have a mental illness *Ndifumana kunzima ukuya ekliniki nje ngoko kumisiwe kuba abantu bazakwazi ukuba mna ndiphazamisekile engqondweni* |  |  |  |  |
| 34 | I think the media has a negative influence on the way people perceive mental illness *Ndicinga ukuba izinto ezisasaza iindaba zinempembelelo engeyiyo kwindlela abantu abajonga ngayo abantu abaphazamisekileyo engqondweni* |  |  |  |  |
| 35 | I find it difficult to take my tablets every day because they remind me that I have a mental illness *Ndifumanisa kunzima ukusela ipilisi zam yonke imihla kuba zindikhumbuza ukuba ndiphazamisekile engqondweni* |  |  |  |  |
